# Uneven distribution of professors and instructors in medical disciplines dealing with the four main chronic non-communicable diseases: the case of the Italian Universities

**DOI:** 10.1186/s40248-017-0108-1

**Published:** 2017-11-02

**Authors:** Giovanni Viegi, Stefano Centanni, Francesco Blasi

**Affiliations:** 1 National Research Council (CNR) Institute of Biomedicine and Molecular Immunology (IBIM), Via Ugo La Malfa 153, 90146 Palermo, Italy; 2 Respiratory Unit, ASST Santi Paolo e Carlo Department of Health Sciences, University of Milan, Milan, Italy; 3 Department of Pathophysiology and Transplantation, Università degli Studi di Milano, Milan, Italy; 4 Internal Medicine Department, Respiratory Unit and Regional Adult Cystic Fibrosis Center, IRCCS Fondazione Cà Granda Ospedale Maggiore Policlinico, Milan, Italy

**Keywords:** Italian universities, Main chronic non-communicable diseases, Professors and instructors, Respiratory medicine

## Abstract

**Background:**

Chronic (non-communicable) diseases (NCD) -- principally cardiovascular diseases, cancer, chronic respiratory diseases, and diabetes -- are leading causes of death and disability. There is the need to adopt a core University curriculum which let students be taught by teachers who are experts of the four main NCD, for reaching the public health goals proposed by the UN and the WHO.

**Methods:**

Our aim was to assess whether all medical students, regardless of the Italian university of enrolment, have an equal opportunity to be educated by an expert teacher in each of the four NCD. We have used the search engine http://cercauniversita.cineca.it/php5/docenti/cerca.php.

**Results:**

In January 2016, for each of the 43 universities with a school of medicine, we have assessed the presence of professors / instructors for each of the four academic disciplines corresponding to the four NCD: a) Respiratory medicine; b) Cardiovascular medicine; c) Oncology; d) Endocrinology. Comparing university personnel between Respiratory medicine and each of the other NCD academic sectors, there were negative differences, much wider with the sector Cardiovascular medicine, regarding individual (number of professors/instructors) and collective indicators (number of Universities with various kinds of professors/instructors).

**Conclusions:**

Both national societies and ERS should promote periodic analyses of the academic situation of respiratory medicine in the European countries for advocating the EU in order to have recommendations/suggestions for the Member States to get the proper recognition of respiratory medicine, at the same level as the other disciplines involved in preventing and managing the four main NCD.

## Background

In 2007 Beaglehole et al. [1] launched a call to action for prevention of chronic diseases. They stated that “chronic (non-communicable) diseases (NCD) -- principally cardiovascular diseases, cancer, chronic respiratory diseases, and diabetes -- are leading causes of death and disability but are surprisingly neglected elements of the global-health agenda. They are underappreciated as development issues and underestimated as diseases with profound economic effects.” Their article opened a series of papers in *The Lancet* providing evidence that the goal for prevention and control of chronic diseases is not only possible but also realistic.

Subsequently, *The Lancet* has continued to publish papers related to the Global Burden of Disease Study [2, 3], which have confirmed the importance of the approach by Beaglehole et al. [1].

On September 19–20, 2011, the United Nations hosted a high-level meeting of the General Assembly on the prevention and control of non-communicable diseases which ended with the adoption of an important political declaration on NCD [4].

On May 27, 2013, the Sixty-sixth World Health Assembly discussing the item “Followup to the Political Declaration of the High-level Meeting of the General Assembly on the Prevention and Control of Non-communicable Diseases” [5] decided to endorse the World Health Organization (WHO) Global Action Plan for the Prevention and Control of NCDs 2013–2020 [6]. Such a report identifies the four main NCD (cardio-vascular diseases, cancer, diabetes and chronic respiratory diseases), for which voluntary global targets for prevention are established. Such targets are proposed to be reached in 2020, for mortality, morbidity and avoidable risk factors.

Among the principles on which actions are based, there is the formulation of “evidence – based” strategies. Among the objectives, it is to be mentioned the objective 5: to promote and support national capacity for high-quality research and development for the prevention and control of non-communicable diseases.

Thus, for the Universities with the School of Medicine a core clinical curriculum is needed to be adopted to let students be taught by teachers who are experts of the four main NCD, in order to educate health personnel whose competences are adequate to help governments reaching the public health goals proposed by the UN and the WHO.

Our aim was to assess whether, in agreement with the principle of equality stated by the article 3 of the Constitution of the Republic of Italy [7], all medical students, regardless of the Italian university where they are enrolled, have an equal opportunity to be educated by an expert teacher in each of the four NCD.

## Methods

We have consulted the web site of the Ministry of Education, University and Research (MIUR). We have used the search engine http://cercauniversita.cineca.it/php5/docenti/cerca.php.

In January 2016, through that search engine, for each of the 43 universities with a school of medicine, we have assessed the presence of professors / instructors (in Italian “ricercatori”) for each of the four academic disciplines corresponding to the four NCD:

- Respiratory medicine (in Italian “*MED 10 - Malattie dell’Apparato Respiratorio*”);
- Cardiovascular medicine (in Italian “*MED 11 - Malattie dell’apparato cardiovascolare*”);
- Oncology (in Italian *“MED 06 - Oncologia Medica”*);
- Endocrinology (in Italian *“MED 13 – Endocrinologia”*).

The web site of the Agency assessing University and Research Systems (in Italian *“Agenzia Nazionale di Valutazione del Sistema Universitario e della Ricerca – ANVUR”)* was visited to check job position and possible recruitment as full professor of those who passed on December 16, 2013 the National Scientific Qualification Exams (in Italian *“Abilitazione scientifica nazionale”*) in the scientific sector 06/D1 (comprising Diseases of the Cardiovascular System and Diseases of the Respiratory System) (expiring on December 16, 2019).

## Results

As regards Respiratory medicine (*MED 10 - Malattie dell’Apparato Respiratorio)* (Table 1), the total number of professors/instructors was 109 on January 7, 2016. Universities with Full Professors were 20, corresponding to 46.51%. Conversely, Universities with no Professor/Instructor of Pulmonology were 12, corresponding to 27.91%.

**Table 1:** Composition of university personnel in NCD academic sectors in the 43 Italian Universities with School of Medicine

	MED 10	MED11	MED06	MED 13
Full professors	25	41	27	46
Full professors (temporary)	1	2	1	0
Associate professors	39	80	37	82
Instructors	37	97	52	76
Instructors (temporary)	7	19	16	18
TOTAL	109	239	133	222
Universities with at least one full professor	20 (46.51%)	35 (81.40%)	23 (53.49%)	29 (67.44%)
Universities with only associate professors	10 (23.26%)	5 (11.63%)	12 (27.91%)	9 (20.93%)
Universities with only instructors	1 (2.33%)	1 (2.33%)	1 (2.33%)	1 (2.33%)
Universities with NO professor / instructor	12 (27.91%)	2 (4.65%)	7 (16.30%)	4 (9.30%)

As regards Cardiovascular medicine (*MED 11 - Malattie dell’apparato cardiovascolare)* (Table 1), the total number of professors/instructors was 239 on January 7, 2016. Universities with Full Professors were 35, corresponding to 81.40%. Conversely, Universities with no Professor/Instructor of Cardiology were 2, corresponding to 4.65%.

As regards Oncology (*MED 06 - Oncologia Medica)* (Table 1), the total number of professors/instructors was 133 on January 8, 2016. Universities with Full Professors were 23, corresponding to 53.49%. Conversely, Universities with no Professor/Instructor of Oncology were 7, corresponding to 16.30%.

As regards Endocrinology (*MED 13 – Endocrinologia)* (Table 1), the total number of professors / instructors was 222 on January 13, 2016. Universities with Full Professors were 29, corresponding to 67.44%. Conversely, Universities with no Professor/Instructor of Endocrinology were 4, corresponding to 9.30%.

Comparing university personnel between Respiratory medicine (*MED 10 - Malattie dell’Apparato Respiratorio)* and each of the other NCD academic sectors in the 43 Italian Universities with School of Medicine (Table 2), there were negative differences, much wider with the sector Cardiovascular medicine (*MED 11 - Malattie dell’apparato cardiovascolare)*, regarding individual (number of professors / instructors) and collective indicators (number of Universities with various kinds of professors/instructors). The only parameter which showed a positive difference is represented by the number of Universities with NO professor/instructor.

**Table 2:** Differences of university personnel between MED 10 and each of the other NCD academic sectors in the 43 Italian Universities with School of Medicine

	MED11	MED06	MED 13
Full professors	−16	−2	−21
Full professors (temporary)	−1	=	+1
Associate professors	−41	+2	−43
Instructors	−60	−15	−39
Instructors (temporary)	−12	−9	−11
TOTAL	−120	−24	−113
Universities with at least one full professor	−15	−3	−9
Universities with only associate professors	+5	−2	+1
Universities with only instructors	=	=	=
Universities with NO professor / instructor	+10	+5	+8

At last, we updated on January 26, 2016 the job positions for those who passed on December 16, 2013 the National Scientific Qualification Exams as full professor (in Italian *“Abilitazione scientifica nazionale”)* in the scientific sector 06/D1 (comprising Diseases of the Cardiovascular System and Diseases of the Respiratory System) (expiring on December 16, 2019) (Table 3). Among the 24 experts of the sector Cardiovascular medicine (*MED 11 - Malattie dell’apparato cardiovascolare)*, 7 had been appointed by the Universities as Full Professors (29.17%). Conversely, among the 10 experts of the sector Respiratory medicine (*MED 10 - Malattie dell’Apparato Respiratorio)*, none had been appointed by the Universities as Full Professor (0%). It is of note that two of them had meanwhile retired.

**Table 3:** Update at January 26, 2016 of job positions for those who passed on December 16, 2013 the National Scientific Qualification Exams as full professor (in Italian “*Abilitazione scientifica nazionale*”) in the scientific sector 06/D1 (comprising Diseases of the Cardiovascular System and Diseases of the Respiratory System) (expiring on December 16, 2019)

	MED 10	MED11
Full professors	0	7
Full professors (temporary)	1	0
Associate professors	5	11
Associate professors (retired)	1	0
CNR Director of Research	1	1
Head Hospital Unit	1	0
Head Hospital Unit (retired)	1	0
Data not found	0	5
TOTAL	10	24

Considering the distribution of specialists in the four most populated European countries [8] (Table 4), in 2014 Italy generally showed the largest number of specialists with regard to France, Germany, UK. When stratifying by specialty, Cardiology was the most frequent one in each country, followed by either Oncology in Italy and Germany or Pulmonology in France and UK. The least frequent specialty was Endocrinology in each country. The largest difference among pairs of specialties was shown by Italy for the comparison Pulmonology vs Cardiology (−15.87 per 100,000 inhabitants).

**Table 4:** Physicians by medical specialty in the four most populated EU countries. Number per hundred thousand inhabitants. Year 2014

	Pulmonology (P)	Cardiology (C)	Δ (P-C)	Oncology (O)	Δ (P-O)	Endocrinology (E)	Δ (P-E)
Italy	6.27	22.14	- 15.87	7.32	- 1.05	3.81	2.46
France	4.46	10.41	- 5.95	1.35	3.11	2.76	1.70
Germany	2.71	8.34	- 5.63	3.39	- 0.68	0.76	1.95
UK	4.05	5.22	- 1.17	3.88	0.17	2.45	1.60

Source Eurostat [9] http://appsso.eurostat.ec.europa.eu/nui/show.do?dataset=hlth_rs_spec&lang=en

## Discussion

The analyses of the MIUR data show a worrisome situation for the academic sector Respiratory medicine (*MED 10 - Malattie dell’Apparato Respiratorio)* in Italy in early 2016.

Such a discipline ranked last among the academic sectors with the task of studying and teaching the knowledge of the four main NCD for either total number of both professors/instructors or Universities with at least one full professor. The latter parameter corresponds to less than 50% of the Universities with the School of Medicine. On the other hand, the academic sector Respiratory medicine (*MED 10 - Malattie dell’Apparato Respiratorio)* ranked first for a very negative indicator: Universities with NO professor/instructor amounted to about 30%.

Such discouraging situation is in evident contrast with the principle of equality stated by the article 3 of the Constitution of the Republic of Italy [7]. In fact, it produces impediments, for the medical students enrolled in the Universities where pulmonology is not taught by pulmonologists, to acquire a deep knowledge of respiratory medicine. As a consequence, there may be a lack of awareness about the importance of chronic respiratory diseases, which, in turn, may be a severe obstacle for Italy to meet the objectives proposed by the WHO Global Action Plan for the Prevention and Control of NCDs 2013–2020 [6].

In addition, the paucity of full professors of respiratory medicine in Italy may hamper the Italian participation to the calls for research proposals issued by the 8^th^ EU Framework Programme on Research & Development (R&D) (Horizon 2020), notwithstanding the big effort made by the European Respiratory Society to convince the European Commission to introduce respiratory diseases among the R&D priorities [9].

This situation can also have severe consequences on the sustainability and dissemination of specialization schools in Respiratory medicine throughout Italy.

In Italy there are 21 Post Graduate schools in Respiratory medicine distributed throughout the Country with some criticisms in densely populated Regions such as Piedmont (over 4 million inhabitants), where just one post graduate School is active at the University of Turin (4 specialists per year), and Veneto (over 6 million people together with Trentino Alto Adige and Friuli Venezia Giulia) with just one School at the University of Padua (4 specialists per year). In the academic year 2015/16, the expected allowed number for pulmonologists in training (jointly calculated by Ministry of Health and Ministry of University and Scientific Research) was the lowest among the four considered specialties: 127, and only 97 (78%) training contracts were actually disbursed. The corresponding numbers for the other three medical specialties were: 320 (82.5%) contracts actually activated out of 388 expected for Cardiology; 148 (80%) vs 185 for Oncology and 86 (80%) vs 105 for Endocrinology.

Regional data should, of course, be updated and planned upon the real epidemiological needs of the population, avoiding reductions of individual specialist hospital services.

MIUR should therefore expand the funds to let Italian universities appoint new full professors in the academic sector Respiratory medicine (*MED 10-Malattie dell’Apparato Respiratorio)* in order to make students achieve a thorough knowledge of pulmonology.

The feasibility of such an objective is demonstrated by the situation of Cardiovascular medicine (*MED 11 - Malattie dell’apparato cardiovascolare)*, academic sector for which the teaching shortage was limited to 18.60% (considering the lack of full professors) and to 4.65% (considering the lack of both professors and instructors).

If the current academic situation will not be changing in the next years, it could negatively influence the production of specialists in respiratory medicine which are already much less than the specialists in cardiology in the four most populated EU countries.

## Conclusions

It would be worthwhile that both national societies and ERS promote periodic analyses (for instance, every three-five years) of the academic situation of respiratory medicine in the European countries. Such evidence could be used for advocating the EU in order to have recommendations/suggestions for the Member States. The goal should be to get the proper recognition of respiratory medicine, at the same level as the other disciplines involved in preventing and managing the four main NCD.
